# *In vitro* effects of fetal rat cerebrospinal fluid on viability and neuronal differentiation of PC12 cells

**DOI:** 10.1186/2045-8118-9-8

**Published:** 2012-06-28

**Authors:** Mohammad Nabiuni, Javad Rasouli, Kazem Parivar, Homa M Kochesfehani, Saeid Irian, Jaleel A Miyan

**Affiliations:** 1 Department of Biology, Tarbiat Moallem University, Tehran, Iran; 2 Department of Biology, Science and Research Branch, Islamic Azad University, Tehran, Iran; 3 Faculty of Life sciences, The University of Manchester, AV Hill Building, Oxford Road, Manchester, M13 9PT, UK

**Keywords:** Cerebrospinal fluid, PC12 cells, Neuronal differentiation, Fetal rat

## Abstract

**Background:**

Fetal cerebrospinal fluid (CSF) contains many neurotrophic and growth factors and has been shown to be capable of supporting viability, proliferation and differentiation of primary cortical progenitor cells. Rat pheochromocytoma PC12 cells have been widely used as an *in vitro* model of neuronal differentiation since they differentiate into sympathetic neuron-like cells in response to growth factors. This study aimed to establish whether PC12 cells were responsive to fetal CSF and therefore whether they might be used to investigate CSF physiology in a stable cell line lacking the time-specific response patterns of primary cells previously described.

**Methods:**

*In vitro* assays of viability, proliferation and differentiation were carried out after incubation of PC12 cells in media with and without addition of fetal rat CSF. An MTT tetrazolium assay was used to assess cell viability and/or cell proliferation. Expression of neural differentiation markers (MAP-2 and β-III tubulin) was determined by immunocytochemistry. Formation and growth of neurites was measured by image analysis.

**Results:**

PC12 cells differentiate into neuronal cell types when exposed to bFGF. Viability and cell proliferation of PC12 cells cultured in CSF-supplemented medium from E18 rat fetuses were significantly elevated relative to the control group. Neuronal-like outgrowths from cells appeared following the application of bFGF or CSF from E17 and E19 fetuses but not E18 or E20 CSF. Beta-III tubulin was expressed in PC12 cells cultured in any media except that supplemented with E18 CSF. MAP-2 expression was found in control cultures and in those with E17 and E19 CSF. MAP2 was located in neurites except in E17 CSF when the whole cell was positive.

**Conclusions:**

Fetal rat CSF supports viability and stimulates proliferation and neurogenic differentiation of PC12 cells in an age-dependent way, suggesting that CSF composition changes with age. This feature may be important *in vivo* for the promotion of normal brain development. There were significant differences in the effects on PC12 cells compared to primary cortical cells. This suggests there is an interaction *in vivo* between developmental stage of cells and the composition of CSF. The data presented here support an important, perhaps driving role for CSF composition, specifically neurotrophic factors, in neuronal survival, proliferation and differentiation. The effects of CSF on PC12 cells can thus be used to further investigate the role of CSF in driving development without the confounding issues of using primary cells.

## Background

The central nervous system develops around a fluid filled tube, the neural tube. Initially the tube forms around amniotic fluid which is then modified by secretions from a structure in the mesencephalon which transports blood components into the neural tube fluid [1]. This has been shown to form a powerful growth medium, called neural tube fluid, or embryonic cerebrospinal fluid (ECSF), for neural stem cells, stimulating proliferation and differentiation in the developing brain stem and spinal cord [2-5]. The cerebral cortex develops much later, the initiation of which coincides with a change in the fluid source to the choroid plexus (CP) as well as an increase in fluid volume and a consequential need for exit from the tube and drainage [6,7]. Subsequently, cerebrospinal fluid (CSF) is secreted by the CP, highly vascularised secretory epithelial structures in the lateral, third and fourth ventricles. During development CSF is rich in protein in contrast to the low protein content in normal adults [8,9]. It is secreted from the initial stages of cortical development and continues to be secreted for the entire life of the individual [10]. Previously, CSF was considered to be a fluid with simple physiological and mechanical functions, but it is becoming increasingly clear that CSF plays critical roles in complicated brain physiology, most especially during development, driving the functions of neural stem cells [2,11-19]. Abnormalities in the CSF system or CSF composition are associated with distinct neurological conditions and global developmental defects/deficiencies [20-26].

CSF is the major element forming the external environment for germinal matrix, stem and progenitor cells of the developing cortex, containing a high concentration of cytokines, growth factors and other proteins secreted by the choroid plexus, and which acts as a growth medium for brain development [1,12,27-29]. Although there is some discussion about the timing of formation and the effectiveness of the different barrier systems within the brain, it is accepted that the ependymal layer forms during late cortical development and that neural stem cells are thus in direct contact with CSF during most of the developmental period [30-33]. The path that CSF follows is a one-way flow from the lateral ventricle, through the third ventricle into the cerebral aqueduct to enter the fourth ventricle where it exits the brain into the surrounding subarachnoid space. The fluid then drains via arachnoid villi into the superior sagittal sinus and/or facial lymphatics [6,34-39], although the latter route may not be present until late in development, in the post natal brain [37]. During this process CSF carries signals derived from different sites within the flow pathway to downstream targets [40]. Previous studies have shown that an obstruction in the fluid pathway results in fluid composition changes that arrest cortical development through a cell cycle blockage [23,24]. This was shown to be due to inhibition of 10-formyl tetrahydrofolate dehydrogenase secretion from cells in the ventricular zone [26]. CSF from different ages of normal fetal development was shown to affect proliferation of primary cortical cells in an age-dependent manner [19]. Because the data also showed an age-dependent response of primary cells, we have now investigated the effect of ECSF on *in vitro* cultures of the PC12 cell line.

PC12 cells are frequently used as an *in vitro* model for neuronal differentiation. These cells differentiate into dopaminergic neurons when cultured with certain growth factors, including nerve growth factor (NGF), basic fibroblastic growth factor (b-FGF), insulin growth factor-I (IGF-I), glial cell line-derived neurotrophic factor (GDNF), epidermal growth factor (EGF) and transforming growth factor-α (TGF-α) [41-44]. It seemed reasonable to use these cells to investigate the effects of developmental CSF without the confounding effects of age-dependent responses of primary cells. The aim of the present study was to investigate the effects of prenatal CSF from various gestational ages on the survival, proliferation and differentiation of PC12 cells.

## Methods

### Animals

Wistar rats were bred in house in the research facility of the Department of Biology, Tarbiat Moallem University following ethical review of the project by the animal use committees of both The University of Manchester and Tarbiat Moallem University. They were kept in large rat boxes at constant temperature and 12hour light/dark cycle with free access to food and water. Individual male and female rats were paired in mating cages and checked regularly for the presence of a vaginal plug which was taken as an indication of successful mating and the day noted as embryonic day 0 (E0). Embryonic age was calculated from that day. At a particular time point pregnant dams were euthanized by cervical dislocation, the uterus rapidly removed onto ice and fetuses dissected out onto ice. Each pregnant dam usually produced between 10–15 fetuses.

### Collection of CSF samples

CSF was collected from the cisterna magna of rat fetuses at E17, E18, E19, and E20 using glass micropipettes and capillary action without aspiration. Aspiration invariably resulted in bleeding and contamination of the samples. Fetuses were positioned with heads flexed down onto the chest to allow penetration into the cisternal cavity through the skin and underlying muscle. Samples containing undesirable blood contamination, visualised as a pink colour in the fluid, caused by damaging a blood vessel within the cisternal cavity, were discarded. All samples were collected into sterile microtubes and centrifuged at 14,000 rpm to remove cells or debris from the fluid, and the supernatant was transferred into another sterile tube. These samples were stored at −80°C until use. The volume of CSF collected from each fetus by this method was between 5 and 50 μl and samples were pooled for each experiment. At least three litters provided six independent pooled samples (half litter per pooled sample) for each age of CSF tested.

### Total protein analysis

Total protein concentration in each pooled CSF sample was determined by the Bio-Rad protein assay (Bio-Rad Laboratories, Hercules, CA, USA), based on the Bradford dye procedure. The absorbance of samples was measured at 595 nm wavelength. Each pooled sample was analysed for each age tested.

### PC12 cell culture and *in vitro* tests

PC12 cells were cultured in RPMI1640 medium (Gibco, Life Technologies Corporation) containing 10% fetal bovine serum, 100 U/ml penicillin and 100 mg/ml streptomycin. The cultures were kept in a humidified incubator with 5% CO_2_ and maintained at 37°C. PC12 culture medium was refreshed in a 2/3 ratio every 2 days. For tests, 100 μl of cells at 4 × 10^4^ cells/ml (4000 cells/well) in RPMI1640 medium were plated into poly-D-lysine coated 96-well plates and cultured in a RPMI1640 medium without serum for 24 hours and then supplemented with CSF (E17-20) (10% v/v) or bFGF (10 ng/ml) for 7 days. bFGF was used as a positive promoter of PC12 cell proliferation and differentiation into neuronal phenotypes to compare to the effects of CSF. After one week, cells were photographed and then prepared for morphological examination and immunocytochemical staining. Three wells were used for each pooled CSF sample giving a total of at least nine wells per CSF age tested. An additional three wells per pooled sample were used for the MTT assay. Cells were photographed at the end of each experiment using phase contrast optics. Cells were then fixed in 4% paraformaldehyde and immunostained for β-tubulin and MAP2 using monoclonal antibodies (Abcam, Cambridge, UK), visualised with FITC conjugated goat-anti mouse secondary antibodies and photographed using fluorescence microscopy. Controls were stained with vehicle solution without the primary antibody. At least six wells were stained for both β-tubulin and MAP2 expression and at least three wells were used as negative controls.

### Measurement of neurite length

For each culture condition, cells in individual wells were photographed with phase-contrast optics (Olympus, Tokyo, Japan) to visualize outgrowths from the cells. Measurements were made using ImageJ software (NIH). A neurite was counted when a cellular process was longer than the diameter of the cell body. The average length of neurites was calculated from measurements of 10 cells in each of 6 wells for each age of CSF tested.

#### Cell viability assay

Cell viability and/or proliferation was quantitatively determined by the MTT method using a colorimetric assay at the end of 7 days in control medium or medium containing CSF or bFGF. MTT (3-(4,5-Dimethylthiazol-2-yl)-2,5-diphenyltetrazolium bromide), is a yellow tetrazolium dye that responds to metabolic activity. Reductase enzymes in living cells reduce MTT from a pale yellow colour to dark blue formazan crystals. Cells were plated at 4000 cells per well as described above. In separate experiments cells were plated at 2000 cells per well with no difference in proliferation or differentiation results (data not shown). At 24 h, prior to addition of CSF or bFGF control plates were analysed for starting number of cells. Experimental and control media plates were left for a further 7 days and then analysed for proliferation. Wells were incubated with MTT (5 mg/mL in PBS) for 3 h at 37°C. In order to make formazan crystals soluble, 0.04 N HCl, prepared in isopropranolol, was added. The absorbance of the formazan product was determined at a wavelength of 570 nm using a plate reader.

### Immunocytochemistry

For immunocytochemistry, after three washes with PBS for 5 min, cells were fixed in 4% paraformaldehyde in PBS for 15 min, permeabilized with 0.1% Triton X-100 for 30 min at room temperature and subsequently blocked with 5% BSA in TPBS (Tween 20 in PBS) for 1 h at room temperature. Cells were incubated at 4°C overnight in the presence of either anti-beta III tubulin mAb (1:50 dilution) or anti-MAP2 mAb (1:50 dilution). The following day, after three washes with TPBS, FITC-conjugated goat anti-mouse IgG (1:250 dilution; Sigma-Aldrich, Poole, UK) was added at room temperature for 1 hr. Cells were then washed and cellular nuclei were counterstained with propidium iodide (Sigma-Aldrich). Photomicrographs were taken with a florescence microscope (Olympus, Tokyo, Japan).

### Statistical analysis

All values are expressed as mean ± standard error of the mean (SEM). Statistical analysis was performed using the one-way ANOVA and Kruskal-Wallis test, and significance was accepted for *p* values of *<*0.05.

## Results

### Total protein concentration

**Figure 1:**
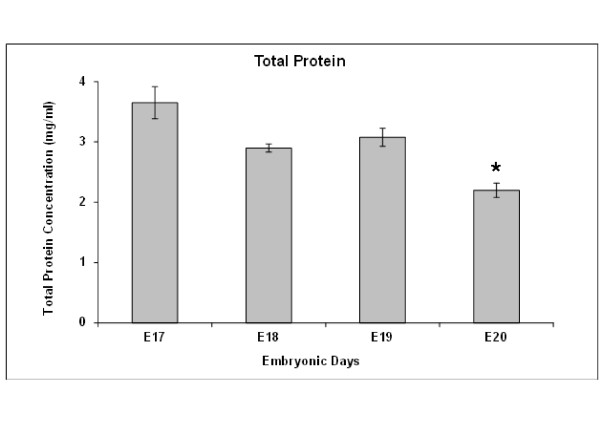
**Total protein content of fetal rat CSF.** Histogram of total protein concentration in pooled samples of cerebrospinal fluid (CSF) from rat fetuses at days E17 to E20. The data shown are mean ± SEM, n = 5 at each age. There was a significant difference between E17 and E20 ECSF protein content p <0.05).

CSF of rat fetuses aged E17 had a mean total protein concentration of 3.65 ± 0.26 mg/ml, which was significantly higher than that of E20 CSF (2.19 ± 0.12 mg/ml, *p* < 0.05, Figure 1). Protein concentrations at E18 and E19 were intermediate at 2.90 and 3.08 mg/ml, respectively.

### Effects of embryonic CSF (ECSF) on the viability and/or proliferation of PC12 cells

**Figure 2:**
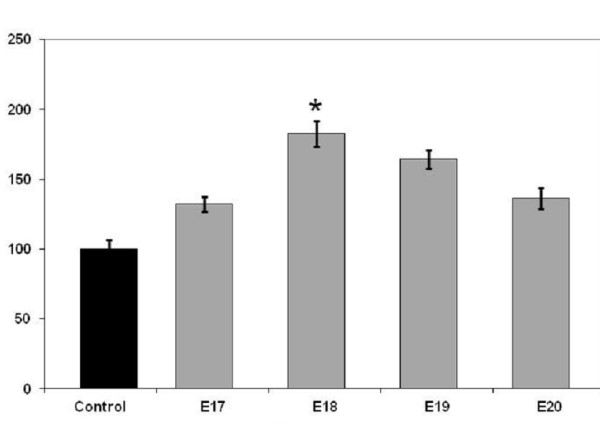
**Survival and/or proliferation of PC12 cells after culture with fetal rat CSF.** Reduction of MTT (3-(4,5-Dimethylthiazol-2-yl)-2,5-diphenyltetrazolium bromide) measured colourimetrically by the absorbance of formazan product. Cells were treated with CSF at different gestational ages (E17-E20) and measured after 7 days in culture. Results are expressed as a percentage of control levels (cultures without added CSF). All cultures with added CSF had higher viability and this was significant with E18 CSF. Data are mean ± SEM, n =9, *; *p* <0.05 compared to controls.

**Figure 3:**
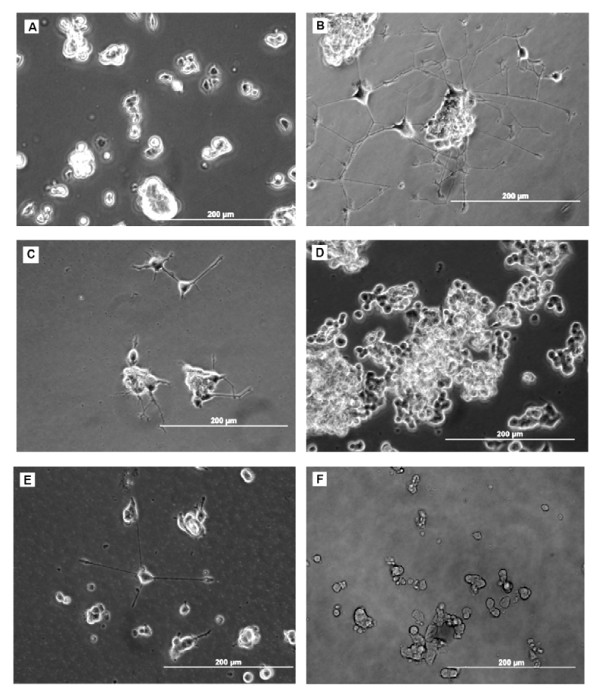
**PC12 cells after 7 days in culture photographed with phase-contrast optics.A:** control culture, **B:** culture with b-FGF, **C:** culture with added CSF-E17, **D:** with CSF-E18, **E:** with CSF-E19 and **F:** with CSF-E20. PC12 cells cultured with ECSF from E17 and E19, and with b-FGF showed neurite outgrowth and morphological differentiation, whereas cells cultured with E-CSF from E18, E20, and the control group (A) did not show any significant differentiation.

**Figure 4:**
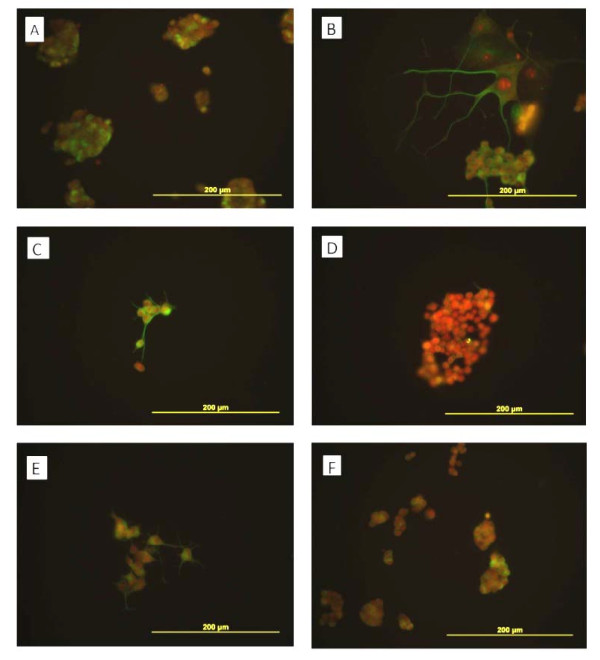
**Beta III tubulin expression (green) in PC12 cells counterstained with propidium iodide (red).** Beta III tubulin is expressed in PC12 cells cultured in normal media (**A**), media with b-FGF (10 ng/ml) (**B**), or with E-CSF from E17 (**C**), E19 (**E**) or E20 (**F**) but not in cells cultured with E18 CSF (**D**).

**Figure 5:**
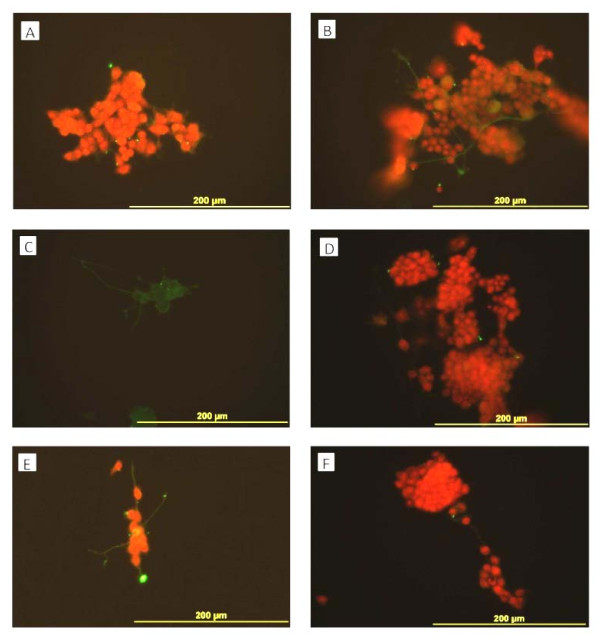
**MAP2 expression (green) in PC12 cells counterstained with propidium iodide (red).** MAP2 expression is shown in PC12 cells cultured with CSF supplemented medium from E17 (**C**), in the neurites of cells in E19 (**E**) and b-FGF (10 ng/ml) (**B**) but not in cells cultured with CSF from E18 (**D**), E20 (**F**), or in control media (**A**).

Figure 2 shows the absorbance of formazan produced by cells treated for 7 days in culture with CSF from different gestational ages. Compared to controls, a higher absorbance (*p* < 0.05) was obtained when cells were cultured with the medium supplemented with CSF of any age indicating greater viability of cells compared with that in media alone. E18 CSF gave a significant increase in viability over that seen in media alone which fits with our previous data from primary cortical cells where E18 and E19 CSF gave increased proliferation of E20 cells [24]. For the PC12 cells cultured with CSF, there was greater clumping of cells suggestive of greater stimulation of cell-cell adhesion rather than cell-substrate adhesion (Figures 3,4 and 5). This requires further investigation.

### CSF induces neuronal differentiation in PC12 cells

Phase-contrast images of cultured PC12 cells are shown in Figure 3. Compared to control medium (Figure 3A), enhanced neurite outgrowth and morphological differentiation occurred with cells incubated with bFGF (Figure 3B), and in the presence of the medium supplemented with CSF-E17 (Figure 3C) and E19 (Figure 3E). Little or no morphological differentiation was detected in the control medium (Figure 3A), with CSF-E18 (Figure 3D), or with CSF-E20 (Figure 3F). However, with CSF-E18, PC12 cells showed more proliferation. Clumping of cells was seen with CSF and also with b-FGF, suggestive of cell-cell interactions rather than “simple” clumping due to a lack of initial separation of cells in plating. We utilized the growth factor bFGF (10 ng/ml) to compare its effects on differentiation with those of ECSF treatment.

### Immunocytochemical characteristics of differentiated PC12 cells

We found beta III tubulin expression in PC12 cells grown in control media as well as in media supplemented with E17, E19 and E20 CSF but not in E18 CSF (Figure 4) By contrast we found little evidence of MAP2 expression in PC12 cells (Figure 5) except in the neurites of cells cultured with bFGF or CSF from E17 or E19 but not in control media or in CSF from E18 or E20. Interestingly, cells cultured in E17 CSF showed expression in neurites and in their cell bodies (Figure 5c).

#### Measurement of neurite outgrowth

**Figure 6:**
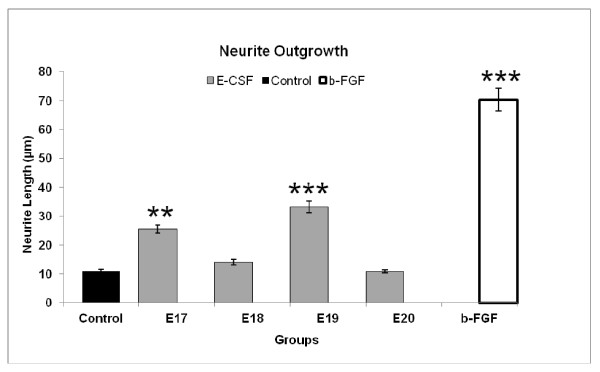
**Neurite growth in PC12 cell cultures.** Length of neurites of PC12 cells cultured with E-CSF-supplemented medium and b-FGF (10 ng/ml). PC12 cells were grown on 96-well cell culture plates for 7 days. Neurite length was measured as described. Data are mean ± SEM, n = 9, **; *p* < 0.01 and ***; *p* < 0.001 compared to control culture.

The average neurite outgrowth of cells was significantly greater than controls when cultured in the presence of b-FGF (*p* < 0.001), or CSF from E17 (*p* < 0.01) and E19 (*p* < 0.001) for 7 days (Figure 6). It was not increased over controls in CSF from E18 or E20 though there was a non-significant increase in E18 CSF. In addition, and as shown in Figure 3, the density and length of neurites was much greater in b-FGF treated cells than in CSF (E17 and E19) treated cells.

## Discussion

The present study has shown that CSF from rat fetuses at different developmental ages (E17-E20) has a high protein concentration of around 3 mg/ml that declined to 2 mg/ml by E20. CSF when added to cultures of PC12 cells had different effects on survival, proliferation and neuronal differentiation depending on age. In contrast to the control groups, samples of CSF at all ages tested, gave a greater formazan absorbance reading indicating improved cell survival and/or proliferation. This was only significant with E18 CSF indicating a greater effect of CSF at this age. Interestingly we previously showed a greater proliferation of primary rat cortical cells in both E19 and E18 CSF over both controls and E17 or E20 CSF [19]. The significant and most surprising result in the current study is the failure of PC12 cells to respond to E18 CSF with any measurable differentiation in either the MAP2 or beta III tubulin study, even though E18 CSF stimulated greater proliferation. These findings would fit with a three stage process, initial production of preplate neurons at E17, high rates of proliferation within the ventricular zone at E18, and migration of immature neurons and differentiation of migrated neurons within the cortical plate over E18-E20 [45,46]. The difference between the effects of PC12 cells and primary cortical cells indicate a possible difference between the pre-programmed development of the *in vivo* neural stem/progenitor cells and the driving force of CSF composition in isolation.

In our experiments, we used cisternal CSF which is likely to contain both proliferation, differentiation and migration signals as it contains all the additions to CSF that are made as the fluid passes through the ventricles. It would of great value to test ventricular CSF and compare its effects since our previous arguments suggest that this may only contain proliferation and possibly differentiation signals but not migration signals [6,7,23,25]. Thus, one outcome of this study is that the parallel use of primary cortical cells and PC12 cells can help to elucidate the varying roles of developmental time-dependent programming of cells *versus* the contributions made by the changes in CSF composition with age.

Almost 75% of CSF is secreted *in vivo* by the CP located in the lateral ventricles with an additional 10% and 5% secreted by the CP in the third and fourth ventricles respectively [47,48]. Additional components are added to the ventricular CSF from the interstitial fluid of the brain parenchyma and from specific organs including the circumventricular organs. The most studied of these is the subcommissural organ which has been shown to be vital for particular physiological functions as well as keeping the CSF pathways open [49-54]. Recent research demonstrates wa significant, if not major role for CSF in the survival, proliferation, migration and differentiation of neural stem/progenitor cells [5,11,12,19,55]. Where problems exist in the CSF system, whether in flow or composition, this developmental program is adversely affected in ways that interfere in normal development [4,20,34,49,56-61]. The most critical constituents of CSF are its protein components, the quality and quantity of which change during CNS development [62-66]. About 20% of CSF proteins are derived from CNS, neurons, glial and leptomeningeal cells, whereas the remaining 80% originate from the blood or are synthesized by the CP [30,67]. The CP synthesizes and secretes many proteins, including various growth factors and neurotrophic factors into the CSF [30,67]. These proteins are carried by CSF bulk flow and provide the developing brain with trophic support for cell survival and neurogenesis [68]. The utilization of neutralizing antibodies against growth factors in CSF showed that blocking of FGF2 in chick ECSF reduces cell proliferation, cell viability and neurogenesis in chick neuroepithelium [69], while the thickness of the neuroepithelium and neuronal precursor proliferation decreased after anti-NGF antibody was injected into the fluid cavity of developing chick brain [70,71]. The current study provides a parallel cell line-based analysis system to that of primary brain cells to investigate the role of CSF. This approach is important as the interaction between primary cell age and CSF age has already been demonstrated [19] while the isolated effect of CSF on a cell line might not expose all the interactions. The *in vitro* survival, proliferation and neuronal differentiation of PC12 cells are dependent on certain growth factors which must also be present in the CSF to elicit the effects observed in this study. The evidence from previous studies indicates that understanding the detailed role of CSF in development, function and pathophysiology of the brain will be one key productive area to promote normal development and to develop strategies and treatments to prevent abnormal development and neuropathological conditions. Isolating CSF components responsible for these different effects can be achieved using the parallel approach we propose. In our recent work we have described a unique folate handling system serving the developing cerebral cortex that operates through the CSF [26]. Obstruction to CSF flow or drainage results in a failure of cortical cells to release 10-formyl tetrahydrofolate, which we believe acts as a folate binding and transporter protein in CSF, and to an arrest in cell cycle and consequential deficient cortical development [23,24,26]. Folate supply to the cerebral cortex can be affected independently of supply to the rest of the CNS and body and result in various cerebral folate deficiencies underlying a variety of neurological conditions that can be alleviated by specific folate supplements [20,22,26,57,61,72-81]. In addition to this folate supply to the developing cortex, there is a complex mix of growth factors and other important proteins in developmental CSF that are affected by CSF drainage and obstruction which remain to be tested for direct effects on the process of cortical development (unpublished data).

## Conclusions

This study has shown that CSF from fetal rat brains of different gestational ages can promote the survival, proliferation and differentiation of PC12 cells in an age dependent manner. Significant differences exist between the response of PC12 cells compared to primary fetal cortical cells perhaps indicating an interaction between the programming of primary cells and age-dependent composition differences in CSF in the latter case. The use of PC12 cells in future studies of CSF physiology may therefore allow the identification of CSF factors effecting cell physiology in isolation of primary cell, *in vivo* programming.

## Abbreviations

ANOVA: analysis of variance; BSA: Bovine serum albumin; CNS: Central Nervous System; CP: choroid plexus; CSF: cerebrospinal fluid; ECSF: Embryonic cerebrospinal Fluid; FITC: fluorescein isothiocyanate; IgG: Immunoglobulin G; MAP: mitogen-activated protein; MTT: 3-(4,5-dimethylthiazol-2-yl)-2,5-diphenyltetrazolium bromide; PBS: phosphate buffered saline; PC: pheochromocytoma.

## Competing interests

None of the authors have any competing interests.

## Authors’ contributions

MN and JAM conceived and designed the experiments. JR, KP, HMK and SI carried out the experiments. All authors were involved in writing and editing the manuscript and preparation of figures. All authors have read and approved the final version of the manuscript.
